# Higher underestimation of tumour size post-neoadjuvant chemotherapy with breast magnetic resonance imaging (MRI)—A concordance comparison cohort analysis

**DOI:** 10.1371/journal.pone.0222917

**Published:** 2019-10-10

**Authors:** Wen-Pei Wu, Hwa-Koon Wu, Chih-Jung Chen, Chih-Wie Lee, Shou-Tung Chen, Dar-Ren Chen, Chen-Te Chou, Chi Wei Mok, Hung-Wen Lai

**Affiliations:** 1 Department of Diagnostic Radiology, Changhua Christian Hospital, Changhua, Taiwan; 2 School of Medicine, Kaohusiung Medical University, Kaohsiung, Taiwan; 3 Department of Pathology, Taichung Veterans General Hospital, Taichung, Taiwan; 4 School of Medicine, Chung Shan Medical University, Taichung, Taiwan; 5 Department of Medical Technology, Jen-Teh Junior College of Medicine, Nursing and Management, Miaoli, Taiwan; 6 Division of General Surgery, Department of Surgery, Changhua Christian Hospital, Changhua, Taiwan; 7 Comprehensive Breast Cancer Center, Department of Surgery, Changhua Christian Hospital, Changhua, Taiwan; 8 Division of Breast Surgery, Department of Surgery, Changi General Hospital, Singapore; 9 Endoscopic & Oncoplastic Breast Surgery Center, Department of Surgery, Changhua Christian Hospital, Changhua, Taiwan; 10 School of Medicine, National Yang Ming University, Taipei, Taiwan; Medical University of Vienna, AUSTRIA

## Abstract

**Objectives:**

The aim of this study was to evaluate the diagnostic accuracy of breast MRI for detecting residual tumor and the tumor size whether it would be affected after neoadjuvant chemotherapy.

**Methods:**

Total 109 patients with NAC and 682 patients without NAC were included in this retrospective study. Measurement of the largest diameter of tumors at pathology was chosen as gold standard and compared with preoperative breast MRI. A concordance threshold of ±25% of maximal tumor size was used. The accuracy of MRI was graded as concordant, underestimation, or overestimation rate. Further subgroup analysis with tumor stages, histologic subgroups and intrinsic subtypes was performed.

**Results:**

The post-NAC MRI was associated with 92.5% sensitivity, 55.2% specificity, 85.1% positive predictive value, 72.7% negative predictive value, and overall 82.6% accuracy for detecting residual tumor. In determining tumor size, the overall concordance rates of the non-NAC group and the NAC group were 43.5% and 41.3%, respectively (p = 0.678). But the overestimation rate and underestimation rate were 26.6% and 32.1% for NAC group, and 52.9% and 3.5% for the non-NAC group (p<0.001). While in the subgroups analysis, the concordance rate of the NAC group (26.7%) was lower than that of the non-NAC group (82.1%) at T3 stage (p<0.001). There were no statistically significant differences between different tumor histologic subgroups and intrinsic subtypes.

**Conclusions:**

The overall accuracy of MRI in predicting tumor size was not affected by NAC; however, it tends to underestimate tumor size after NAC, especially in patients with T3 lesions and above.

## Introduction

Neoadjuvant chemotherapy (NAC) has been shown to effectively downstage breast cancer, increase resectability, and facilitate breast-conserving surgery (BCS)[1–4]. Currently, there is growing evidence that a pathologic complete response (pCR) following NAC is an indicator for better prognostic outcome, including local regional recurrence and disease-free or overall survival[2, 5–8]. Adequate assessment of therapeutic response and obtaining a precise estimate of the residual tumor field size were found to be key elements in the prediction of patients’ prognosis and for planning of surgical strategy[9–11].

As magnetic resonance imaging (MRI) is widely adopted in clinical use, breast MRI is a promising imaging modality for evaluation of breast cancer before[12–18] or after neoadjuvant chemotherapy[4, 11, 19–25]. The tumor enhancement depends on certain biological effects of the tumors such as the vascularity and capillary permeability[26, 27], which might be affected after NAC[9, 28]. Currently, few studies evaluated the diagnostic accuracy of breast MRI in the determination of breast tumor size after NAC. We hypothesized that the diagnostic performance of breast MRI in the determination of tumor size in terms of concordance (concordant, overestimation, or underestimation rate) might be affected by NAC.

The aim of the current study was to evaluate the diagnostic accuracy of breast MRI for detecting residual tumor and the tumor size whether it would be affected by NAC. Factors affecting the discrepancy between the accuracy of MRI-predicted tumor size and histopathology-derived tumor size were also compared and analyzed according to different tumor stages, histologic subgroups and intrinsic subtypes.

## Materials and methods

### Patients

**Fig 1:**
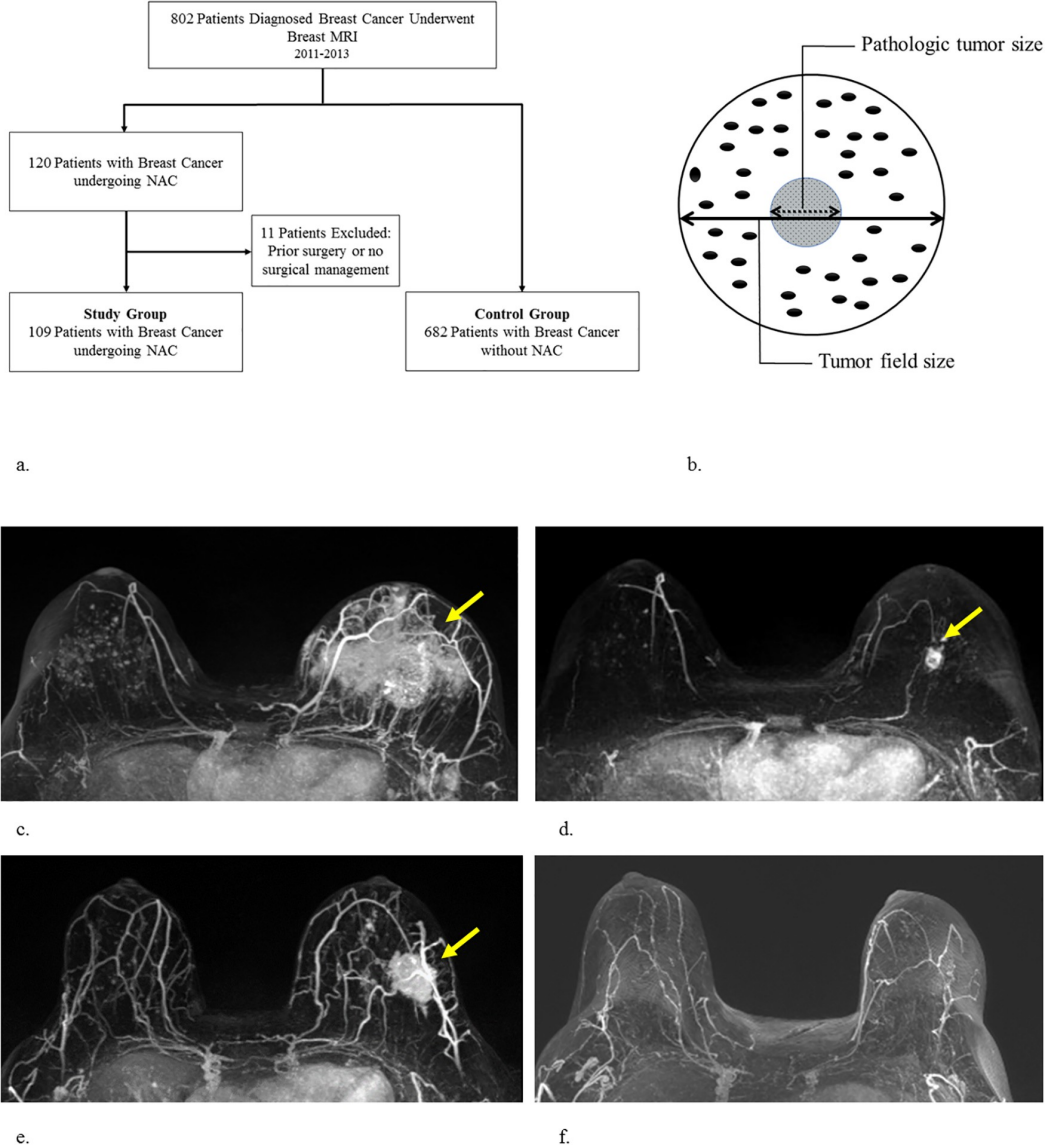
Study design, illustration of tumor size, and MRI interpretation. **(a) Study design. (b) The definitions of tumor field size and pathologic tumor size. (c,d) Luminal type A cancer of left breast**. Baseline MRI scan before (c) and after (d) NAC treatment. After NAC treatment, the mass had decreased in size to 2.2cm. Response was classified as non-complete response. The final pathologic analysis disclosed a measurement of 1.3cm. Therefore, MRI overestimated the tumor size of the residual tumor. **(e,f) Triple negative tumor of left breast**. Baseline MRI scan before (e) and after the NAC treatment was completed (f). Following NAC, pCR was suggested by both MRI and final pathology. The tumor sizes of breast MRI and pathology are concordant.

A retrospective study was conducted in which patients who received NAC and subsequent definitive cancer surgery were selected from the breast cancer MRI database at Changhua Christian Hospital (CCH). Patients were required to have at least 2 MRI (a baseline MRI, which was performed before NAC, and another post-NAC MRI, which was performed before surgical resection) during January 2011 through December 2013. To evaluate for the diagnostic accuracy of breast MRI in patient groups with and without NAC, the diagnostic performance of MRI after NAC was compared with another cohort of patients without NAC as the control group, which was reported in our previous study[12]. The study design is shown in Fig 1a.

Following the exclusion of patients who did not meet the inclusion criteria, the NAC group comprised of 109 patients with baseline and post-NAC MRI who received definitive surgery at CCH. The control group included 682 breast cancer patients who did not receive NAC and obtained pre-operative breast MRI contributed non-NAC group (Fig 1a). Clinical and pathologic data of these patients were collected through chart review of medical, surgical, and pathologic records. This study was approved by the Institutional Review Board (IRB) of Changhua Christian Hospital (IRB number #140404). Consent waiver was performed for this study due to the concealment of any patient identification.

### Histologic evaluation: Definition of tumor size and pathologic complete response

Pre-NAC biopsy and post-NAC surgery specimens were stained with hematoxylin & eosin (H&E) and immunohistochemistry (IHC) stain. Post-NAC tumor size, histologic type, and treatment response (pCR rate) were obtained from the pathologic report. The results of pathologic histologic subtypes were classified according to the content of carcinoma in situ lesions, infiltrating ductal carcinoma (IDC), infiltrating lobular carcinoma (ILC), or other subtypes[12]. The intrinsic subtypes were classified according to consensus guidelines[29, 30]. Pathologic tumor size for invasive cancer was defined as the largest diameter in centimeters. Tumor field size was defined as the largest diameter of invasive and carcinoma in situ lesions[12] (Fig 1b). The pathologic complete response (pCR) was defined as the absence of residual invasive carcinoma and regional lymph nodes (ypT0/Tis, N0)[31].

### Concordance between imaging and pathologic results

In our previous study[12], we showed that using a concordance definition with ±25% maximal tumor size instead of ±0.5cm as a cutoff point was a more objective criterion for estimating the MRI-derived breast cancer tumor size. The measurement of the residual tumor size by post-NAC MRI was compared with the pathologic-derived tumor size for concordance. Two different cutoff points (±25% maximal tumor size or ±0.5cm) were used to test for concordance. When MRI-derived tumor size was compared with histopathologically determined tumor size, the difference was within ±25% (or ± 0.5 cm), and the result was thus deemed to be concordant. Results measuring < -25% (or -0.5cm) were deemed to indicate underestimation of tumor size, and results > 25% (or 0.5 cm) were deemed to indicate overestimation of tumor size.

### MRI measurement of tumor size and assessment of tumor response

The MRI was performed with a Siemens (Verio) 3.0 Tesla MR machine with a dedicated 16-channel bilateral breast coils for all patients. All patients were imaged in the prone position. MRI protocols included the following: axial T1-weighted, axial T2-weighted fat-suppressed images. Dynamic MRI included 6 sets of images using a three-dimensional T1-weighted fat-suppressed breath-hold (VIBE) sequence performed before and after a bolus injection of gadobenate dimeglumine (Multihance^®^, Bracco, Milan, Italy), 0.1mmmol/kg at a flow rate of 2 ml/s using an MR-compatible power injector followed by a 20-ml saline flush, with a 60-s interval of each phase dynamic images. After the dynamic studies, delayed three-dimensional T1-weighted turbo spin echo images were acquired.

Measurement of tumor size by post-NAC MRI was performed using a commercially available MRI computer aid diagnosis (CAD) system with computer-based tumor segmentation by DynaCAD Version 2.1 (Invivo, Gainesville, FL). In accordance with the response evaluation criteria in solid tumors (RECIST)[32], MRI tumor size was measured using the longest tumor diameter. For multiple tumors, the greatest diameter of tumor size was measured. We defined residual tumor as an abnormally enhancing area at the previous tumor site according to pre-NAC MRI, regardless of the presence of washout kinetics (Fig 1d). Complete response (CR) was diagnosed when no enhancement or faint enhancement equal to the background normal breast tissue was present (Fig 1f). The MRI examinations were interpreted before the definitive breast surgery by one experienced, board-certified breast radiologist (HKW) with 15 years’ experience reading breast MRI. The reader was not blinded to carcinoma location, patient history, or prior examinations.

### Statistical analysis

Data are expressed as mean ± standard deviation (SD) for continuous variables. Categorical data are expressed as numbers and percentages. Independent *t* tests are used for the comparison of continuous variables. Categorical variables are normally tested by the χ2 test when appropriate. The relations between MRI size and pathology include Spearman’s rank correlation coefficient (ρ) and Bland-Altman plots including 95% confidence intervals and limits of agreement (LOA)[33]. All p values are two-tailed; a p value of less than 0.05 is considered to indicate statistical significance. All statistical analyses was performed by SPSS 22.0 software (IBM, Armonk, NY, USA).

## Results

### Accuracy of MRI in predicting response to NAC

The demographic data of the study population is shown in Table 1. Among the 109 patients who received NAC, 29 (26.6%) patients experienced a pCR, and 80 (73.4%) had residual cancer in the final pathologic examinations. Of the 22 patients who were considered to have had a complete response at the post-NAC MRI, 16 patients (72.7%) had a complete response in the pathologic review. Of the 87 patients judged to have residual tumor according to the post-NAC MRI, 74 (85.1%) patients were found to have residual tumor in the final pathology report. The diagnostic accuracy of post-NAC MRI in predicting residual tumor was obtained as follows: sensitivity 92.5% (95% confidence interval (CI): 86.7%-98.3%), specificity 55.2% (95% CI: 37.1%-73.3%), positive predictive value (PPV) 85.1% (95% CI: 77.6%-92.5%), negative predictive value (NPV) 72.7% (95% CI: 54.1%-91.3%), and accuracy 82.6% (95% CI: 75.4%-89.7%) (Table 2).

**Table 1:** Demographic data of patients who did not receive neoadjuvant chemotherapy (NAC) and those who received NAC.

	Study Group Post-NAC Patients	Control Group Non-NAC Patients	*p* value
	N = 109 patients	N = 682 patients	
**Gender (Female: Male)**	109:0 (100:0%)	681:1 (99.9: 0.1%)	1.00
**Age (year, mean)**	47.8 ± 10.1 (28–68)	53.1 ± 10.7 (20–90)	< 0.001
**MRI tumor size (mean)**	1.88 ± 1.94 (0–9.6) cm	3.64 ± 1.8 (0.7–11) cm	< 0.001
**Pathology tumor size (mean)**	1.51± 2.12 (0–11) cm	2.3 ± 1.6 (0.1–10) cm	< 0.001
**Tumor field size (Invasive + non-invasive)**	2.72± 2.68 (0–11) cm	2.78 ± 1.7 (0.1–11.5) cm	0.76
**Tumor field size—pathologic tumor size (mean)**	1.20 ± 2.39 (-4.7–10.3) cm	0.49 ± 1.34 (0–11.2) cm	< 0.001
**Tumor field size—pathologic tumor size (%)**			0.24
** Difference<0**	11 (10.1%)		
** Difference = 0**	48 (44.0%)	500 (73.3%)	
** Difference≤0.5 cm**	6 (5.5%)	60 (8.8%)	
** 0.5< Difference≤1 cm**	9 (8.3%)	25 (3.7%)	
** 1< Difference≤2 cm**	11 (10.1%)	36 (5.3%)	
** Difference > 2 cm**	24 (22.0%)	61 (8.9%)	
**MRI-Pathologic tumor size**	0.37± 1.67 cm (-8.2–6.1)	1.34 ± 1.63 cm (-4.5–9.2)	< 0.001
**MRI-Tumor field size**	- 0.84 ± 2.50 cm (-9.3–4.7)	0.85 ± 1.25 cm (-4.5–7.0)	< 0.001
**T stage at presentation (%)**			0.21
** T1 (≦2 cm)**	4 (3.7%)	256 (37.5%)	
** T2 (2–5 cm)**	76 (69.7%)	359 (52.6%)	
** T3 (>5 cm)**	15 (13.8%)	67 (9.8%)	
** T4**	14 (12.8%)	0 (0%)	
**Histologic subgroups**		(n = 666, NA = 16)	0.22
** DCIS**	0 (0%)	101(15.2%)	
** IDC + DCIS**	57 (52.3%)	288(43.2%)	
** IDC**	48 (44.0%)	235(35.3%)	
** ILC**	1 (0.9%)	22(3.3%)	
** Other subtypes**#	3 (2.8%)	20(3%)	
**Intrinsic subtypes**		(n = 680, NA = 2)	0.24
** Luminal A**	31 (28.4%)	285 (41.9%)	
** Luminal B1**	14 (12.8%)	176 (25.8%)	
** Luminal B2**	32 (29.4%)	99 (14.5%)	
** TNBC**	14 (12.8%)	58 (8.5%)	
** HER-2**	18 (17.4%)	62 (9.1%)	
**Chemotherapy regimens**			
** Anthracycline-based chemotherapy**	16 (14.7%)	-	
** Taxane-based chemotherapy**	5 (4.6%)	-	
** Anthracycline + Taxane**	33 (30.3%)	-	
** Taxane + Target therapy (Herceptin and/or Pertuzumab)**	44 (40.4%)	-	
** Others**	11 (10.1%)	-	

^#^ Other subtypes: mucinous carcinoma, papillary carcinoma, metaplastic carcinoma, neuroendocrine cancer, malignant phyllodes tumor. The intrinsic subtypes (Luminal A, Luminal B1, Luminal B2, HER-2, and triple negative breast cancer) were classified according to the expression levels of estrogen receptor (ER), progesterone receptor (PR), HER-2/neu, and Ki-67 results.

**Table 2:** Accuracy of breast MRI for detecting residual breast cancer after neoadjuvant chemotherapy and comparisons with selected studies in the literature.

Study	Year	Journal	Patient no.	Sn	Sp	PPV	NPV	LR+	LR-	Accuracy
Nakamura et al.[34]	2007	Breast Cancer	115	89	48	88	50	1.71	0.22	81.7
Moon et al.[21]	2009	Ann Oncol	195	96	38	90	38	1.55	0.10	88
Straver et al.[22]	2010	Ann Surg	208	78	67	90	44	2.35	0.33	76
Williams et al.[23]	2013	Am J Surg	87	86	77	92	64	3.79	0.18	84
Zhang et al.[24]	2014	J Thorac Dis	114	95	29	80	67	1.33	0.17	78
Ko et al.[25]	2015	Korean J Radiol	174	93	68	91	74	2.88	0.10	61
Bouzon et al.[19]	2016	Radiol Oncol	91	75	79	89	58	3.5	0.32	76
Lai et al. (current)			109	93	55	85	73	2.06	0.14	83

### Assessment of extent of residual malignant disease post-NAC

**Fig 2:**
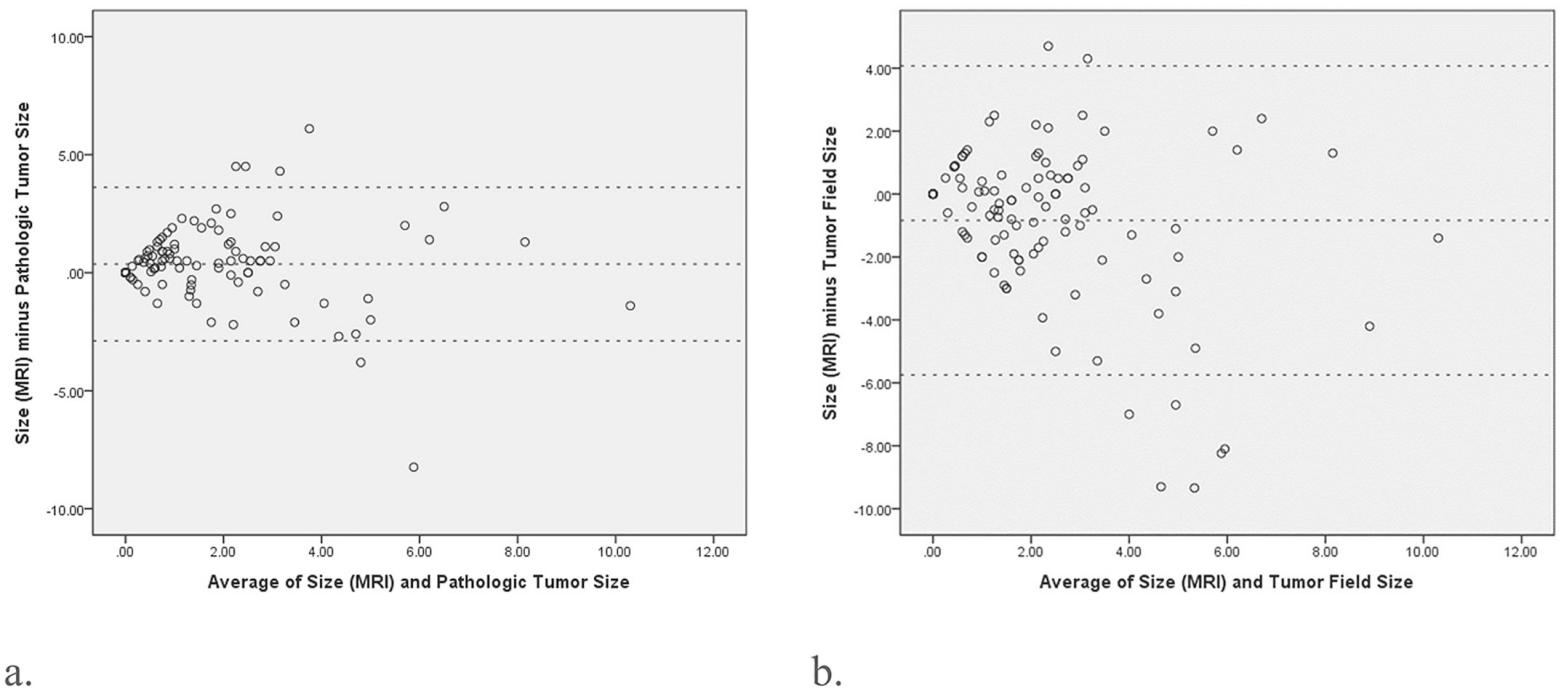
The Bland-Altman plot shows the agreements of the tumor size measurements of MRI scans with pathologic tumor size or tumor field size. **(2a)** For the pathologic tumor size, the limits of agreement (LOA) are -2.9 and 3.6 cm with corresponding 95% confidence interval (mean bias 0.4cm, SD 1.7cm). **(2b)** For the tumor field size, the LOA are -5.7cm and 4.1cm with corresponding 95% confidence interval (mean bias—0.8cm, SD 2.5cm).

The Spearman’s rank correlation coefficient between post-NAC MRI and pathologic tumor size is 0.680 (95% confidence interval (CI): 0.547–0.779, p<0.001), and that between post-NAC MRI and tumor field size is 0.401 (95% CI: 0.221–0.574, p<0.001). The LOA, as assessed using the Bland-Altman technique(38), is shown in Fig 2a and 2b, where wider LOA indicates poor agreement. For the pathologic tumor size, the LOA values are -2.9 and 3.6 cm, with corresponding 95% CI (mean bias 0.4cm, SD 1.7cm); for the tumor field size, the LOAs values are -5.7cm and 4.1cm, with corresponding 95% CI (mean bias -0.8cm, SD 2.5cm), respectively.

### Effect of NAC on the diagnostic accuracy of MRI-predicted tumor size

The diagnostic accuracy of MRI in the prediction of post-NAC tumor size is further analyzed and compared with that of the non-NAC group. The difference between tumor field size and pathologic tumor size is 1.20 ± 2.39 cm in the post-NAC group of patients, compared with 0.49 ± 1.34 in the non-NAC group of patients (Table 1). When tumor field size is used as a reference, the difference between MRI and tumor field size is -0.84 ± 2.50 cm in the post-NAC group of patients, compared with 0.85 ± 1.25 in the non-NAC group of patients. The concordance rates of the non-NAC group and post-NAC group with a 0.5cm cutoff point are 37.5% and 30.3% (p = 0.164), and those with a 25% cutoff point are 43.5% and 41.5%, respectively (p = 0.678, Table 3). When the pathologic tumor size is used as reference, the concordance rates of the non-NAC group and post-NAC group with a 0.5cm cutoff point are 30.2% and 44.0% (p = 0.006), and those with a 25% cutoff point are 33.7% and 37.6%, respectively (p = 0.448, Table 3).

**Table 3:** Analysis of diagnostic accuracy of breast MRI in non-neoadjuvant chemotherapy (non-NAC) group and post-NAC group.

		Concordance	Overestimation	Underestimation	* P value	** P value
**MRI–Pathologic tumor size ± 0.5cm cutoff point**						
Non-NAC Group		206 (30.2%)	453 (66.4%)	23(3.4%)	0.006	<0.001
Post-NAC Group		48 (44.0%)	43 (39.4%)	18 (16.5%)		
**MRI–Pathologic tumor size ± 25% cutoff point**						
Non-NAC Group		230 (33.7%)	437 (64.1%)	15(2.2%)	0.448	<0.001
Post-NAC Group		41 (37.6%)	53 (48.6%)	15 (13.8%)		
**MRI–Tumor field size ± 0.5cm cutoff point**						
Non-NAC Group		256 (37.5%)	384 (56.3%)	42(6.2%)	0.164	<0.001
Post-NAC Group		33 (30.3%)	27 (24.8%)	49 (45.0%)		
**MRI–Tumor field size ± 25% cutoff point**						
Non-NAC Group		297 (43.5%)	361 (52.9%)	24(3.5%)	0.678	<0.001
Post-NAC MRI Group		45 (41.3%)	29 (26.6%)	35 (32.1%)		
**Tumor Stage at Diagnosis**						
** T1**	Non-NAC (n = 256)	54 (21.1%)	198 (77.3%)	4 (1.6%)	0.204^+^	1.000^+^
	Post-NAC (n = 4)	2 (50%)	2 (50%)	0 (0%)		
** T2**	Non-NAC (n = 359)	188 (52.4%)	159 (44.3%)	12 (3.3%)	0.614^+^	<0.001^+^
	Post-NAC (n = 76)	37 (48.7%)	15 (19.7%)	24 (31.6%)		
** T3**	Non-NAC (n = 67)	55 (82.1%)	4 (6%)	8 (11.9%)	<0.001^+^	0.640^+^
	Post-NAC (n = 15)	4 (26.7%)	2 (13.3%)	9 (60%)		
** T4**	Non-NAC (n = 0)	-	-	-	-	-
	Post-NAC (n = 14)	2 (14.3%)	10 (71.4%)	2 (14.3%)		
**Pathologic Tumor Stage**						
** T0 or Tis**	Non-NAC (n = 0)	-	-	-	-	-
	Post-NAC (n = 28)	17 (60.7%)	6 (21.4%)	5 (17.9%)		
** T1**	Non-NAC (n = 256)	54 (21.1%)	198 (77.3%)	4 (1.6%)	0.263^++^	<0.001^++^
	Post-NAC (n = 50)	15 (30%)	15 (30%)	20 (40%)		
** T2**	Non-NAC (n = 359)	188 (52.4%)	159 (44.3%)	12 (3.3%)	1.000^++^	<0.001^++^
	Post-NAC (n = 20)	10 (50%)	4 (20%)	6 (30%)		
** T3**	Non-NAC (n = 67)	55 (82.1%)	4 (6%)	8 (11.9%)	0.002^++^	1.000^++^
	Post-NAC (n = 10)	3 (30%)	3 (30%)	4 (40%)		
** T4**	Non-NAC (n = 0)	-	-	-	-	-
	Post-NAC (n = 1)	-	1 (100%)	-		
**Histology**						
** IDC-DCIS**	Non-NAC (n = 288)	136 (47.2%)	143(49.7%)	9(3.1%)	0.109^†^	<0.001^†^
	Post-NAC (n = 57)	20 (35.1%)	16 (28.1%)	21 (36.8%)		
** IDC**	Non-NAC (n = 235)	97(41.3)	133(56.6)	5(2.1)	0.268^†^	<0.001^†^
	Post-NAC (n = 48)	24 (50%)	11 (22.9%)	13 (27.1%)		
** ILC**	Non-NAC (n = 22)	13 (59.1%)	6 (27.3%)	3 (13.6%)	0.435^†^	-
	Post-NAC (n = 1)	-	1 (100%)	-		
** Others**	Non-NAC (n = 22)	16 (72.7%)	6 (27.3%)	-	0.231^†^	-
	Post-NAC (n = 3)	1 (33.3%)	2 (66.7%)	-		
**Intrinsic Subtypes**						
** Luminal A**	Non-NAC (n = 285)	119 (41.8%)	155 (54.4%)	11(3.9%)	0.707^‡^	<0.001^†^
	Post-NAC (n = 31)	14 (45.2%)	6 (19.4%)	11 (35.5%)		
** Luminal B1**	Non-NAC (n = 176)	86 (48.9%)	83 (47.2%)	7 (4.0%)	0.171^†^	0.002^†^
	Post-NAC (n = 14)	4 (28.6%)	5 (35.7%)	5 (35.7%)		
** Luminal B2**	Non-NAC (n = 99)	50 (50.5%)	48 (48.5%)	1(1.0%)	0.839^†^	<0.001^†^
	Post-NAC (n = 32)	15 (46.9%)	6 (18.8%)	11 (34.4%)		
** TNBC**	Non-NAC (n = 58)	20 (34.5%)	34 (58.6%)	4 (6.9%)	0.138^†^	0.538^†^
	Post-NAC (n = 14)	8 (57.1%)	5 (35.7%)	1 (7.1%)		
** HER-2**	Non-NAC (n = 62)	20 (32.3%)	41 (66.1%)	1 (1.6%)	0.562^†^	<0.001^†^
	Post-NAC (n = 18)	4 (22.2%)	7 (38.9%)	7 (38.9%)		

*P value is calculated by comparing concordance versus non-concordance (overestimation + underestimation)

**P value is calculated using Non-NAC MRI as the reference

Multiple comparisons should be corrected with the Bonferroni corrections. And thus ^+^ P value is required a probability of 0.05/4 = 0.0013 for significance; ^++^ P value is required a probability of 0.05/5 = 0.001 for significance; ^†^ P value is required a probability of 0.05/4 = .0013 for significance; ^†^ P value is required a probability of 0.05/5 = 0.001 for significance.

The overestimation rate and underestimation rate of the post-NAC group with the reference of tumor field size and a 0.5cm cutoff point are 24.8% and 45.0%, in contrast to the non-NAC group, which has rates of 56.3% and 6.2%, respectively (p<0.001). When a 25% cutoff point is used, the overestimation rate and underestimation rate of the post-NAC group are 26.6% and 32.1%, in contrast to the non-NAC group, which has rates of 52.9% and 3.5%, respectively (p<0.001, Table 3).

### The diagnostic accuracy of breast MRI in subgroup analysis

There is no statistically significant difference in the diagnostic accuracy of breast MRI between the NAC and non-NAC groups based on intrinsic subtypes or histologic subgroup analysis (Table 3). However, in the subgroup analysis of tumor stage at diagnosis, the post-NAC MRI had a concordance rate of 26.7% and non-NAC MRI had a concordance rate of 82.1% at T3 stage (p<0.001). Similarly, in the pathologic tumor stage subgroups, non-NAC MRI had a concordance rate of 82.1% and post-NAC MRI had a concordance rate of 30% at T3 stage (p = 0.002). The non-concordance MRI imaging analysis reveals a consistent change from higher percentage of overestimation in the non-NAC MRI group to a higher percentage of underestimation in the post-NAC MRI group across all subgroups of patients (Table 3).

## Discussion

Pre-operative evaluation of therapeutic response is becoming increasingly important and is particularly necessary for patients receiving NAC(3,10,11,15). In the current study, the post-NAC MRI is associated with 93% sensitivity, 55% specificity, 85% PPV, 73% NPV, and overall 83% accuracy in predicting residual tumor in the final pathology examination, and this result is consistent with previously reported studies in the literature (Table 2).

In our study, we found that post-NAC MRI showed a greater correlation with pathologic tumor size (r = 0.669) than with tumor field size (r = 0.451, Fig 2). In our previous study, the pre-operative MRI in patients without NAC showed a higher correlation with tumor field size than with pathologic tumor size(12). This might be due to the fact that pathologic tumor size is defined as the largest invasive cancer in the pathologic examination, while tumor field size includes both invasive and non-invasive components in tumors containing carcinoma in situ lesions (Fig 1b).

We observe that the sensitivity of MRI after NAC for detecting carcinoma in situ components is lower relative to that for detecting invasive components. For patients receiving NAC, the concordance rate of breast MRI is 50% in the subgroup of IDC only lesion, compared to 35.1% in the IDC and DCIS subgroup (Table 3). Moreover, in the subgroup of IDC only, the concordance rate of post-NAC MRI (50%) is higher than that of non-NAC MRI (41.3%), p <0.001, while in the IDC-DCIS subgroup, the concordance rate of post-NAC MRI (35.1%) is lower than that of non-NAC MRI (47.2%), p <0.001. In a previous study on the ability of MRI to predict the pathology size of breast tumors, DCIS histology was recognized as a confounding factor[15]. Marinovich et al. also reported that when residual DCIS was excluded in the pCR definition, an increase in accuracy was found[10]. The efficacy of post-NAC MRI to assess the extent of carcinoma in situ lesions therefore requires further validation.

Few studies have investigated whether NAC would decrease the accuracy of MRI in the prediction of tumor size as it similarly does for SLNB detection rate in patients receiving NAC[30, 35, 36]. In our study, we found that the post-NAC MRI has a comparable concordance rate for predicting tumor size compared with patients without NAC (Table 3). However, when the pathologic tumor size is used as the reference tumor size with a cutoff point of 0.5cm, the concordance rate is significantly higher in the post-NAC group than in the non-NAC group (44% versus 30.2%, p = 0.006, Table 3). As the pathologic tumor size is smaller after NAC treatment compared to the non-NAC group (1.5± 2.1cm versus 2.3 ± 1.6cm), a cutoff point of 0.5cm permits a wider range of error for smaller tumors. Therefore, post-NAC breast MRI would have higher concordance rates than MRI in the non-NAC group by adopting a cutoff point of 0.5cm (44% versus 30.2%), compared to that with a cutoff point of ±25% (37.6% versus 33.7%). These results also support our suggestion to use ±25% maximal tumor size as a cutoff point instead of ±0.5cm[12] as we have demonstrated that it is a more objective criterion to show concordance in tumor size evaluation.

One of the main criticisms of the use of MRI in the pre-operative evaluation of breast cancer is that MRI frequently overestimates tumor size[12, 13] and sometimes leads to increased and unnecessary mastectomy rate[14]. However, in this study, we found that the post-NAC MRI shows a significantly increased rate of underestimation in patients who received NAC. The overestimation rate and underestimation rate of the post-NAC group (using tumor field size as the reference and ±25% as the cutoff point) are 26.6% and 32.1%, in contrast to the non-NAC group, which has rates of 52.9% and 3.5%, respectively (p<0.001). This increased underestimation rate is consistently observed in different tumor stages, histologic subgroups, and intrinsic subtypes (Table 3).

The non-concordance (overestimation + underestimation) rate in the post-NAC MRI group is much higher than that of MRI for patients without NAC in tumors at T3 stage (tumors >5cm) either in the initial clinical stage at diagnosis (73.3% versus 17.9%, p<0.001) or at the pathologic tumor stage after NAC (70% versus 17.9%, p = 0.002, Table 3). The underestimation rate of post-NAC MRI for T3 tumors is between 40% (pathologic tumor stage) and 60% (initial clinical stage). It is important to be aware of the high underestimation rate of the post-NAC MRI for large tumors (> T3) during surgical planning to prevent margin involvement.

The high underestimation rate of the post-NAC MRI for predicting residual disease might have resulted from the microscopically scattered tumor foci and loss of tumor cellularity after NAC[37, 38]. In addition, the reduction of enhancement secondary to the changes in tumor vascularization induced by the anti-angiogenetic effect of chemotherapeutic agents also contributed to the underestimation of post-NAC MRI in the prediction of residual tumor size[39].

To evaluate the efficacy of post-NAC MRI in the prediction of breast cancer tumor size, we collected 109 primary breast cancer patients who received at least two separate sets of breast MRI with detailed pathologic information at a single institution. The results were compared to another cohort of patients, who did not receive pre-operative chemotherapy, to answer an important question: whether NAC would affect the concordance rate of MRI.

There are some limitations in this study related to its retrospective nature and the discrepancy between the numbers of patients in the post-NAC and non-NAC groups. Therefore, not all patients in the study data had been treated with the same standard chemotherapy regimens, which accounted for the heterogeneity among the studies. The breast cancer subtype and treatment regimens might influence the accuracy of breast MRI. However, this study yielded valuable information with respect to the use of breast MRI for diagnosis that has not been reported.

## Conclusion

To our knowledge, this study is the first to report on the diagnostic performance of breast MRI with a direct comparison of patients who did or did not receive NAC. Our results showed that after NAC, the accuracy of breast MRI did not differ if compared with patients who did not receive NAC. However, a shift of non-concordance was observed owing to an overestimation in patients who did not receive NAC as well as an underestimation in patients who received NAC. Radiologists and surgeons should be aware of this change to avoid underestimating the actual size and extent of tumors.

## Supporting information

S1 File Original data for this manuscript. (XLSX)
