# Correction: Higher underestimation of tumour size post-neoadjuvant chemotherapy with breast magnetic resonance imaging (MRI)—A concordance comparison cohort analysis

**DOI:** 10.1371/journal.pone.0233532

**Published:** 2020-05-14

**Authors:** The PLOS ONE Staff

There are errors in the author affiliations. The correct affiliations are as follows: Wen-Pei Wu^1,2^, Hwa-Koon Wu^1^, Chih-Jung Chen^3,4,5^, Chih-Wie Lee^1^, Shou-Tung Chen^6,7^, Dar-Ren Chen^6,7^, Chen-Te Chou^1,2^, Chi Wei Mok^8^, Hung-Wen Lai^2,4,6,7,9,10^

**1** Department of Diagnostic Radiology, Changhua Christian Hospital, Changhua, Taiwan, **2** School of Medicine, Kaohusiung Medical University, Kaohsiung, Taiwan, **3** Department of Pathology, Taichung Veterans General Hospital, Taichung, Taiwan, **4** School of Medicine, Chung Shan Medical University, Taichung, Taiwan, **5** Department of Medical Technology, Jen-Teh Junior College of Medicine, Nursing and Management, Miaoli, Taiwan, **6** Division of General Surgery, Department of Surgery, Changhua Christian Hospital, Changhua, Taiwan, **7** Comprehensive Breast Cancer Center, Department of Surgery, Changhua Christian Hospital, Changhua, Taiwan, **8** Division of Breast Surgery, Department of Surgery, Changi General Hospital, Singapore, **9** Endoscopic & Oncoplastic Breast Surgery Center, Department of Surgery, Changhua Christian Hospital, Changhua, Taiwan, **10** School of Medicine, National Yang Ming University, Taipei, Taiwan

The publisher apologizes for the errors.
